# Forest stand spectrum reconstruction using spectrum spatial feature gathering and multilayer perceptron

**DOI:** 10.3389/fpls.2023.1223366

**Published:** 2023-11-22

**Authors:** Fan Wang, Linghan Song, Xiaojie Liu, Shuangwen Zhong, Jiawei Wang, Yao Zhang, Yun Wu

**Affiliations:** ^1^ College of Forestry, Fujian Agriculture and Forestry University, Fuzhou, Fujian, China; ^2^ University Key Lab for Geomatics Technology and Optimize Resource Utilization in Fujian Province, Fujian Agriculture and Forestry University, Fuzhou, Fujian, China

**Keywords:** UAV-based remote sensing, spectrum-spatial fusion, three-dimensional spectral, deep learning, masson pine forest

## Abstract

**Introduction:**

Three-dimensional spectral distributions of forest stands can provide spatial information on the physiological and biochemical status of forests, which is vital for forest management. However, three-dimensional spectral studies of forest stands are limited.

**Methods:**

In this study, LiDAR and multispectral data were collected from Masson pine stands in southern Fujian Province, China, and a method was proposed for inverting forest spectra using point clouds as a unit. First, multispectral values were mapped to a point cloud, and the isolated forest algorithm combined with K-means clustering was applied to characterize fusion data. Second, five deep learning algorithms were selected for semantic segmentation, and the overall accuracy (oAcc) and mean intersection ratio (mIoU) were used to evaluate the performance of various algorithms on the fusion data set. Third, the semantic segmentation model was used to reconfigure the class 3D spectral distribution, and the model inversion outcomes were evaluated by the peaks and valleys of the curve of the predicted values and distribution gaps.

**Results:**

The results show that the correlations between spectral attributes and between spatial attributes were both greater than 0.98, while the correlation between spectral and spatial attributes was 0.43. The most applicable method was PointMLP, highest oAcc was 0.84, highest mIoU was 0.75, peak interval of the prediction curve tended to be consistent with the true values, and maximum difference between the predicted value and the true value of the point cloud spectrum was 0.83.

**Discussion:**

Experimental data suggested that combining spatial fusion and semantic segmentation effectively inverts three-dimensional spectral information for forest stands. The model could meet the accuracy requirements of local spectral inversion, and the NIR values of stands in different regions were correlated with the vertical height of the canopy and the distance from the tree apex in the region. These findings improve our understanding of the precise three-dimensional spectral distribution of forests, providing a basis for near-earth remote sensing of forests and the estimation of forest stand health.

## 1 Introduction

Forests are an essential component of terrestrial ecosystems(Gordon, 2008; Cao et al., 2022), and play a significant role in carbon storage and oxygen release. They also conserve the biodiversity of plants, animals, and microorganisms (Xue et al., 2021), with trees serving as the foundation. Unmanned aerial vehicles (UAVs) equipped with various sensors have obtained multispectral information on forest stands. UAVs provide a precise scientific reference for monitoring the physiological and biochemical status of small-scale forest stands, which is vital for regional forestry resource management.

Forest stand spectral information could be categorized as two-dimensional (2D) or three-dimensional (3D). 2D spectral information is collected through aerial survey operations by multispectral cameras on UAVs, mainly reliant on a 2D raster to illustrate the horizontal dimensional spectral distribution of forest stands. Multispectral images could store reflectance data for forests, such as the spectral values of the vegetation. This allows precise calculations of the band along with texture characteristics of forest (Li T. et al., 2022; Dong et al., 2019), making it easy to characterize the planar structure that comprises the true forests feature distribution (Gao et al., 2020; Kazi and Bannari, 2022). In addition, multispectral information shows the plane configuration of the authentic forest features, and is crucial in the inversion of remote sensing parameters of small-scale woods (Xie et al., 2017; Zhao et al., 2021; Zhou X, 2021; He et al., 2022). Nevertheless, vegetation reflectance metrics are abundant in 2D spectral data while the spatial dimensionality is still limited. Forests have been studied only for their horizontal multispectral information properties while they were 3D objects, which inescapably leads to a blind spot in the sensing of the spectral composition of crucial forest stand components such as the lower and middle canopy along with understory vegetation, resulting in a challenge to describe the 3D spectral distribution of woody plants.

LiDAR sensors have a strong capacity to penetrate the forest canopy (Dayal et al., 2022), UAV-mounted LiDAR sensors or multispectral LiDAR have often been used to gather 3D spectral information of forest stands (Li., Chen, 2021). The primary advantage of UAV-mounted LiDAR sensors is their ability to describe high-precision 3D structural information of forests in the form of point clouds, which are unfortunately restricted by spectra to the three primary colors (R, G, B). Some studies incorporated geographical coordinates with the three primary colors for point cloud classification and segmentation (Peichao et al., 2022), however this strategy somehow failed to recognize vegetation response in the near-infrared or red-edge bands. Meanwhile, UAV-mounted multispectral LiDAR, typically represented by the Canadian Optech Titan system (Van, 2015) (containing three bands: 532, 1064, and 1550 nm), could demonstrate a comparatively broader range of specular reflection bands. Some researchers performed multispectral point cloud feature classification based on the Titan system(Shi et al., 2021; Yang et al., 2021; Luo et al., 2022). In terms of data quality, the point cloud density obtained via this method (1-6/m^2^)(Ahmed et al., 2019; Wang R. et al., 2022) was approximately one percent of the LiDAR, consequently making it difficult to be applied to targeted spectral detection of small-scale forest fractions.

UAV airborne LiDAR can acquire a vast quantity of precise spatial structural information, while multispectral photogrammetry can collect rich waveband records. Various studies have utilized the benefits of the fusion of UAV image features and spatial features from the LiDAR point cloud, in image pixels or voxel units, for the inversion of the forest structure (Wu et al., 2022; Yan et al., 2023). Several researchers (Caiyun et al., 2013; Wang X. et al., 2021) have used single wood as a unit to obtain structural data. Since the feature level rather than the point cloud level was utilized in these studies, it failed to consider the information expression of the fusion point cloud. Point clouds are 3D point sets that show sparse and irregular distribution (Feng et al., 2023), reflecting the precise depth information from the object surface points to the LiDAR (Zhao, 2022). The point cloud spectral data obtained by the airborne LiDAR sensor were relatively small (R, G, B), and feature-level fusion failed to apply the true 3D spatial distribution properties of the LiDAR point cloud data. The texture of the item and its precise reflectance in horizontal form can be defined through multispectral photographs. A few studies have contemplated fused spectral details as input features directly to point cloud data, which can effectively consider the intricate geometric spatial structure features, then achieve better feature segmentation (Wang L. et al., 2022; Jing, 2021; Xue, 2022). However, most research to date has focused on fused point clouds with spectral details only for the exterior layer, the spectral attributes of the middle and lower canopy remain undemonstrated. LiDAR point cloud data products, present certain challenges to the intelligent interpretation of point cloud data, as the canopy is heavily veiled, the use of proper information mining algorithms will improve analyses of complex point cloud information.

Point cloud deep learning (DL) algorithms continue to change as computer technology advances, and it is common practice to utilize semantic segmentation techniques based on point clouds (Lu J et al., 2023). The PointNet network (Qi et al., 2017b) executed modifications to data in consideration of the point cloud’s disorganized and sparse structure, PointNet++ (Qi et al., 2017a), a traditional MLP network, uses a hierarchical feature extraction strategy. Vector local self-attention represented the Point Transformer’s central idea (Zhao et al., 2020). A network solely employing pure MLP architecture was known as Point MLP (Ma et al., 2022), which refraining from the excessive computation costs created by locally complex tasks. Likewise, the convolution algorithm helped alleviate the computing load. In point cloud segmentation tasks, PointCNN (Li et al., 2018) resolved the issue of giving learned trait weights and arranging characteristics in a predetermined potential order. Point Conv (Wu et al., 2018) expanded dynamic filters into an innovative convolutional execution. Engelmann et al. (2017) proposed a point cloud block grouping process; however, this method and some subsequent methods, such as RSNet (Huang et al., 2018), had difficulty defining the learning context of each point. The above algorithms are based directly on point clouds, with different methods for the acquisition and learning of local feature information (Gu et al.,2020). The efficiency of point cloud segmentation models had become progressively exceptional as DL algorithm technology evolved. Point cloud spectral information could be enlarged and topped up with data fusion processing (Wang et al., 2020; Zouhair et al., 2022), with spectral semantic feature learning and the point cloud fusion dataset, spectral information for the true 3D point cloud can be inverted by DL algorithms, achieving end-to-end content reconstruction of the forest 3D point cloud spectrum.

The spectral information for vegetation can be used to estimate important physiological and biochemical parameters (Zhou and Cao, 2021). Remote sensing data are commonly used to acquire the spectral characteristics of forests, including satellite remote sensing and remote sensing images obtained by the UAV platform (Li L et al, 2022). A series of recent studies have indicated that multispectral satellite remote sensing is widely applicable to forestry work. Feng et al. calculated the remote sensing optical index based on Gaofen-6 and EU Sentinel-2A data to invert the FMC (fuel moisture content) of subtropical forests in Guangdong, China (Feng X. et al., 2022). Qiu et al. identified damage from pine wood nematode disease based on Sentinel-2 and Landsat-8 remote sensing satellite images, with an accuracy of up to 79.3% (Qiu and Zong, 2023). However, satellite remote sensing acquisition is of low resolution. By contrast, several studies suggest that the low resolution of satellite remote sensing data could be solved by using multispectral sensor in UAV, which is widely used for small-scale forest parameter acquisition. Based on multispectral UAV data, Zhao et al. constructed 11 plots for the exponential inversion of forest and grass coverage, with an accuracy of up to 90% (Zhao et al., 2023). Lu et al. estimated the photosynthetic parameters of *Linalum japonicum* based on multispectral UAV remote sensing, with *R* ^2^ values up to 0.788 (Lu X. et al., 2023). However, such studies have a narrow focus on 2D datasets, and it is difficult to obtain 3D spectral parameters for stands by satellite remote sensing or UAV remote sensing. Methods to obtain 3D spectral information on forests are lacking.

In this context, a strategy to assess the stand’s 3D spectral distribution based on point cloud spectrum-spatial fusion with a neural network algorithm was proposed in our study. The specific objective of this study was to invert 3D near-infrared spectral values for forest stands in point cloud units. After the fusion of multispectral image values and point clouds, a semantic segmentation method for point clouds based on feature addition and deep learning models was introduced. Finally, an evaluation experiment was conducted to evaluate the inversion accuracy. The main objectives of our study are as follows.

1) We propose an end-to-end accurate pairing of multispectral data and point cloud spatial data to generate a high-dimensional stand point cloud dataset using the hidden point removal and spatial orientation fusion methods.
2) We establish a coupled isolated forest algorithm along with clustering algorithm and a pre-classifier to enhance the features of the fusion dataset and then test semantic segmentation accuracy and performance of point cloud DL algorithms of different classes (PointNet, Pointnet ++, PointMLP, Point Transformer, and Point Conv).
3) To evaluate the potential of merging DL methods with point cloud fusion in the 3D spectral reconstruction of forest stands and evaluate the spectrum inversion results using spectral frequency distribution graphs.

This study provides a method for the remote sensing inversion of the stereoscopic spectral distribution of stands to a certain extent, with practical implications for monitoring variation in forest stands.

## 2 Materials and methods

### 2.1 The study area

Hetian Town (25°35’-25°46’ N,116°16’-116°30 ‘E), Changting County, Longyan City, Fujian Province, China is the study area. Hetian Town has a subtropical monsoon climate and is located southwest of Fujian Province, it is low-lying, with numerous low mountains and hills scattered throughout the territory, the stand’s basic composition is *Pinus massoniana*, and the zonal vegetation is subtropical evergreen broad-leaved forest (Li T et al., 2022).

### 2.2 Data acquisition

**Figure 1:**
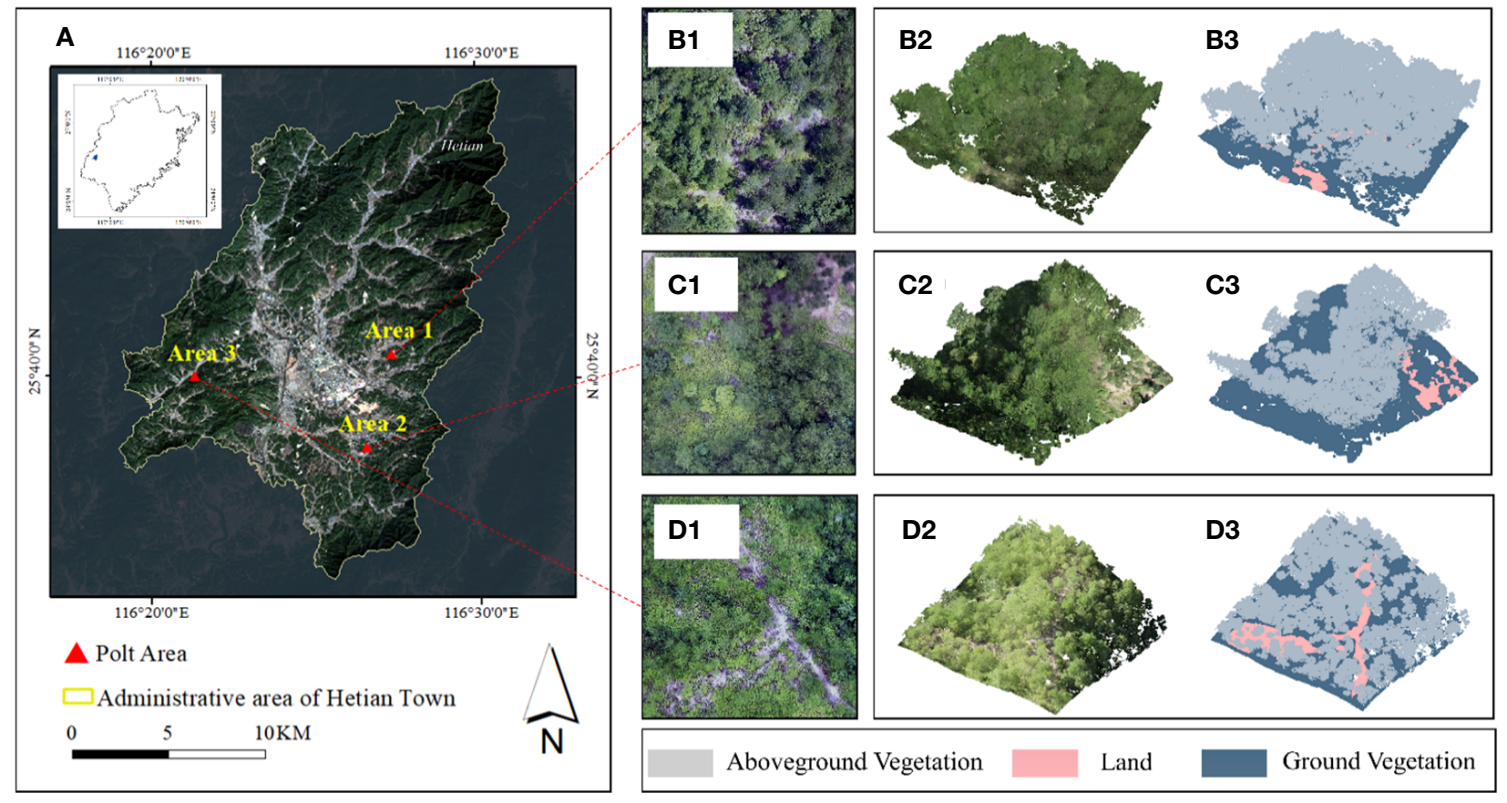
Schematic of the research area. **(A)** Administrative division of Hetian Town. Orthophotos of **(B1)** plot 1, **(C1)** plot 2, and **(D1)** plot 3, and LiDAR data for **(B2)** plot 1, **(C2)** plot 2, and **(D2)** plot 3. Class distributions of **(D1)** plot 1, **(D2)** plot 2, and **(D3)** plot 3.

1) UAV Multispectral Remote Sensing Data Acquisition: This study set three standard plots of *Pinus Massoniana* with a size of 20 m*20 m under the condition of the canopy density gradient. The vegetation distribution of the sample plots is primarily *Smunda* and *Pinus Massoniana*, the proportion of the total area of bare land increases as canopy density decreases (**Figure 1**). To ensure that highly precise standard ground coordinates were obtained, after locating the sample site with DJI UAV high-precision RTK in August 2022, DJI Spirit 4 equipped with the Mica Sense Red Edge multispectral lens was used to collect multispectral orthophotos of the study area on sunny and windless days. DJI Spirit 4 has a positioning system supporting high-precision GNSS and network RTK, with 1 visible light lens and 5 multispectral lenses (blue, green, red, red edge, near-infrared) integrated. The flight altitude was set to 80 m, the flight speed to 3 m/s, and the overlap between the flight heading and the side direction was set to 80% during the acquisition process, we obtained 341 orthoimages to acquire spectral information, the multispectral aerial photographs were radio-metrically corrected, cropped, and stitched using Photo Scan software (http://www.agisoft.cn/) to obtain three parts of orthophotos, each part including R, G, B, NIR, red-edge, and true color, these images were resampled to 0.01 m resolution in ArcMAP 10.2 to ensure the accuracy of the subsequent data fusion.

2) UAV LiDAR Data Acquisition: At the same time, the DJI M300 RTK with L1 laser lens was used to collect LiDAR point cloud data in the same area with a laser spot size of 52 mm*491 mm and a wavelength of 905 nm, the collection mode was waypoint hovering mode during the data collection, the number of echoes was 3, the flight altitude was 80 m, the flight speed was 3 m/s, the pulse emission frequency was 160 kHz, the overlap rate between heading and side direction was 80%, the point density exceeded 600/m^2^ and the scanning angle was ±30°, further to geometric registration, the obtained point cloud data were stored in LAS format, which include 3D coordinates, primary colors, intensity, scanning angle, return-times, and other information. Noise in the point cloud obtained by the airborne laser scanner would influence subsequent data processing; to address this issue, we used Cloud Compare (http://www.cloudcompare.org/) for noise removal. Then, CSF cloth filtering (Wuming et al., 2016) was used to ensure that ground points and above-ground points are separated from the de-noising cloud for pre-classification. The cloud distribution at sample sites is shown in **Table 1**.

**Table 1:** Statistical table of various data of sample set.

	Canopy density	Number of points	Multispectral image Resolution/(M^2^)	Number of points per class		
				tree	land	grass
Plot 1	0.20	267593	0.01	108857	75722	83014
Plot 2	0.35	290446	0.01	95444	97393	97609
Plot 3	0.60	272775	0.01	104402	83474	84899
Total	\	830814	\	308703	256589	265522

### 2.3 Methods

**Figure 2:**
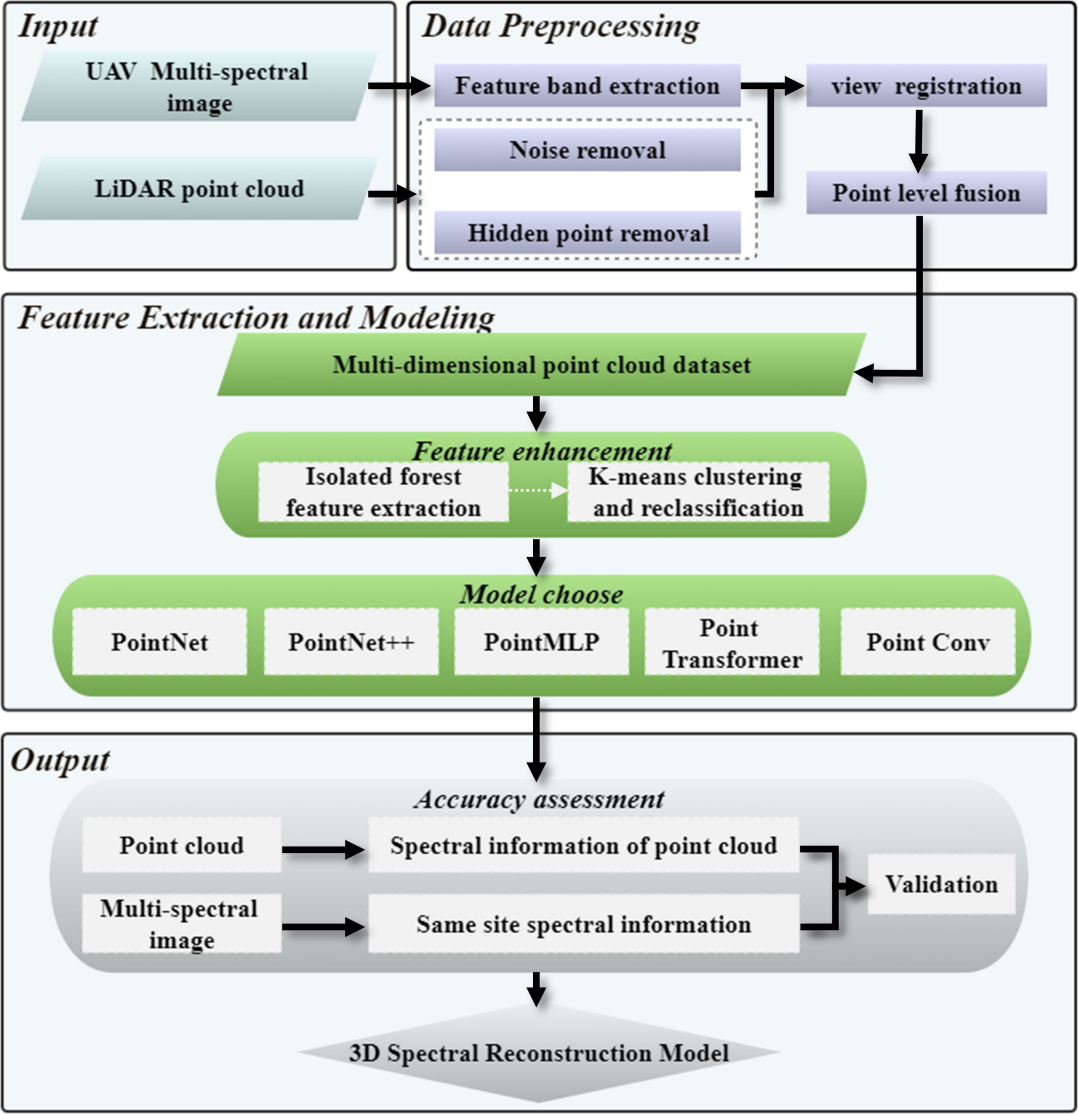
Overall work flowchart.

**Figure 2** provides an overview of the study’s methodological approach. We applied a strategy for reconstructing 3D spectrum data by combining point cloud and multispectral photographic imagery with semantic segmentation of the point cloud. By combining the advantages of 2D spectral information and the 3D spatial structure of the point cloud, semantic segmentation technology was used to produce 3D spectral products of forests.

1) We first extracted the feature bands of UAV multispectral image data, then based on airborne LiDAR point cloud data set, Cloud Compare was used for noise removal and pre-segmentation. We used Open3D for hidden point removal, constructed on perspective and normal vectors. When the UAV multi-spectral sensor collects data, the shooting Angle of the lens is approximately vertical to the ground, and the spectral information of the surface of the ground object is obtained in this way, however, the airborne LiDAR point cloud of UAVs contains all the spatial structure information of both the surface and lower surface objects. Therefore, we can assume that the intersection of UAV multi-spectral sensor and LiDAR is the surface of ground object, to couple the multi-spectral data and LiDAR point cloud data, eliminating the invalid point clouds outside the surface point clouds of ground objects is necessary, only in this way can we construct the point cloud data set of fusion spectrum. Then, the remained point cloud was fused with the spectrum as the prior knowledge of the 3D spectral point cloud, and point level fusion was achieved with spatial orientation as a reference to obtain a point cloud collection containing feature multispectral data.

2) We then combined isolated forest and K-means pre-classification to create sub-classifiers for feature augmentation in response to point cloud collection noise and internal feature difference obscurity. Due to the differences in the acquisition methods for multispectral data and point cloud data, after fusion, the features of the point cloud data set were obscure and the data amount was huge. The focus on isolated forests could eliminate abnormal outliers in large data sets, and K-means clustering could be used to scale the spectral law of point clouds to enhance 3D spectral features.

3) We compared five types of DL algorithms to explore the applicable limitations of point cloud feature data and determine the optimal semantic segmentation architecture based on the dataset after outlier removal and feature extension. The types of deep learning algorithms included typical MLP methods, attention mechanism methods, pure MLP methods, and convolution methods. Different deep learning algorithms could extract different local feature information from point clouds.

4) Finally, we verified the inversion results and developed a 3D spectral inversion model. The spatial distributions of stand spectra before and after inversion were visually evaluated, and the spectral point cloud curves were the drawn for analyses. The original 3D point cloud data were used for point-level spectral inversion. multispectral images in the same region were taken as validation data, and the consistency of the data distribution frequency characteristics in the real 3D perspective was compared to evaluate the accuracy of the model.

The key components of the 3D spectral inversion model included the point-level fusion of the point cloud and multi-spectrum and deep learning semantic segmentation. Point-level semantic fusion ensured the accuracy of prior knowledge in deep learning, where point-level spectral inversion refers to spectral inversion in terms of a 3D point cloud, based on which we can obtain the spectral parameters of each point cloud instead of the 2D image element spectral values, and expand the point cloud spectral information to obtain the 3D forest spectral features.

#### 2.3.1 Feature fusion methods of LiDAR data and multispectral image

1) Hidden Point Removing Methods: The feature fusion work was mainly based on the spatial orientation consistency of the object being detected, approximating high-precision geographic alignment and information mapping, the point cloud needed to be subjected to hidden point removal (HPR)(Katz et al., 2007), a method that removes the internal point cloud that was not visible from any of the aerial survey simulation views of the UAV, using the open3D in python 3.6 environments to complete the data processing, to assure that the point convergence for the fusion task was located in the multispectral camera’s shooting area, the forest exterior point cloud was collected by extracting the feature point cloud. The HPR algorithm works on the following principles: The HPR algorithm was based on the following principles:

a) Mapping: given coordinate system P and C, where viewpoint C is located at the origin, The point set P is drawn inside the coordinate system, and p_i_ is located on the path of monotonically decreasing radioactivity from the origin C, Set a D-dimensional sphere with C as the origin and R as the radius. Through (1), spherical inversion is used to solve the image points reflected from the mapping point set to the outside relative to the sphere’s interior.


(1) $$\hat{p_{i}}=f(p_{i})=p_{i}+2(R-\|p_{i}\|)\frac{p_{i}}{\left\|p_{i}\right\|}$$

where $\hat{p_{i}}$ represents the set of mapping points and R represents the radius.

b) Convex hull reconstruction: For the transformed point cloud and spherical center point, the point existing on the convex hull of the sphere is extracted as the proposed visible point.

2) Spectrum-spatial Data Fusion: The point cloud after the removal of the hidden point was fused with the multispectral image, the fusion was based on the spatial azimuth settlement of the aerial photo and the 3D coordinates of the point cloud. With known orientation elements inside and outside the aerial camera film, the point cloud 3D coordinates were substituted into the co-linear condition equation to calculate the pixel positions of the corresponding 3D points on the image (Zhang et al., 2009), which were then resampled to obtain the near-infrared (NIR) channel grayscale values, in (2): (X, Y, Z) represents the point cloud 3D coordinates, (X_S_, Y_S_, Z_S_) are the three line elements representing the outer orientation elements of the point cloud, (a_1_, b_1_, c_1_, a_2_, b_2_, c_2_, a_3_, b_3_, c_3_) are the parameters of the rotation matrix calculated from the three angular elements of the outer orientation elements. The spatial-spectral matching was completed by locating the 3D coordinate points to specific image element positions in the multispectral photograph, resulting in a point cloud dataset containing (X, Y, Z, R, G, B, NIR) 7-dimensional information, which was used as *a priori* input knowledge for subsequent spatial-spectral internal information perception.


(2) $$\begin{array}{r} x=-f\cdot \frac{a_{1}(X-X_{s})+b_{1}(Y-Y_{s})+c_{1}(Z-Z_{s})}{a_{3}(X-X_{s})+b_{3}(Y-Y_{s})+c_{3}(Z-Z_{s})}\\ y=-f\cdot \frac{a_{2}(X-X_{s})+b_{2}(Y-Y_{s})+c_{3}(Z-Z_{s})}{a_{3}(X-X_{s})+b_{3}(Y-Y_{s})+c_{3}(Z-Z_{s})} \end{array}$$

#### 2.3.2 Feature enhancement based on fusion dataset

**Figure 3:**
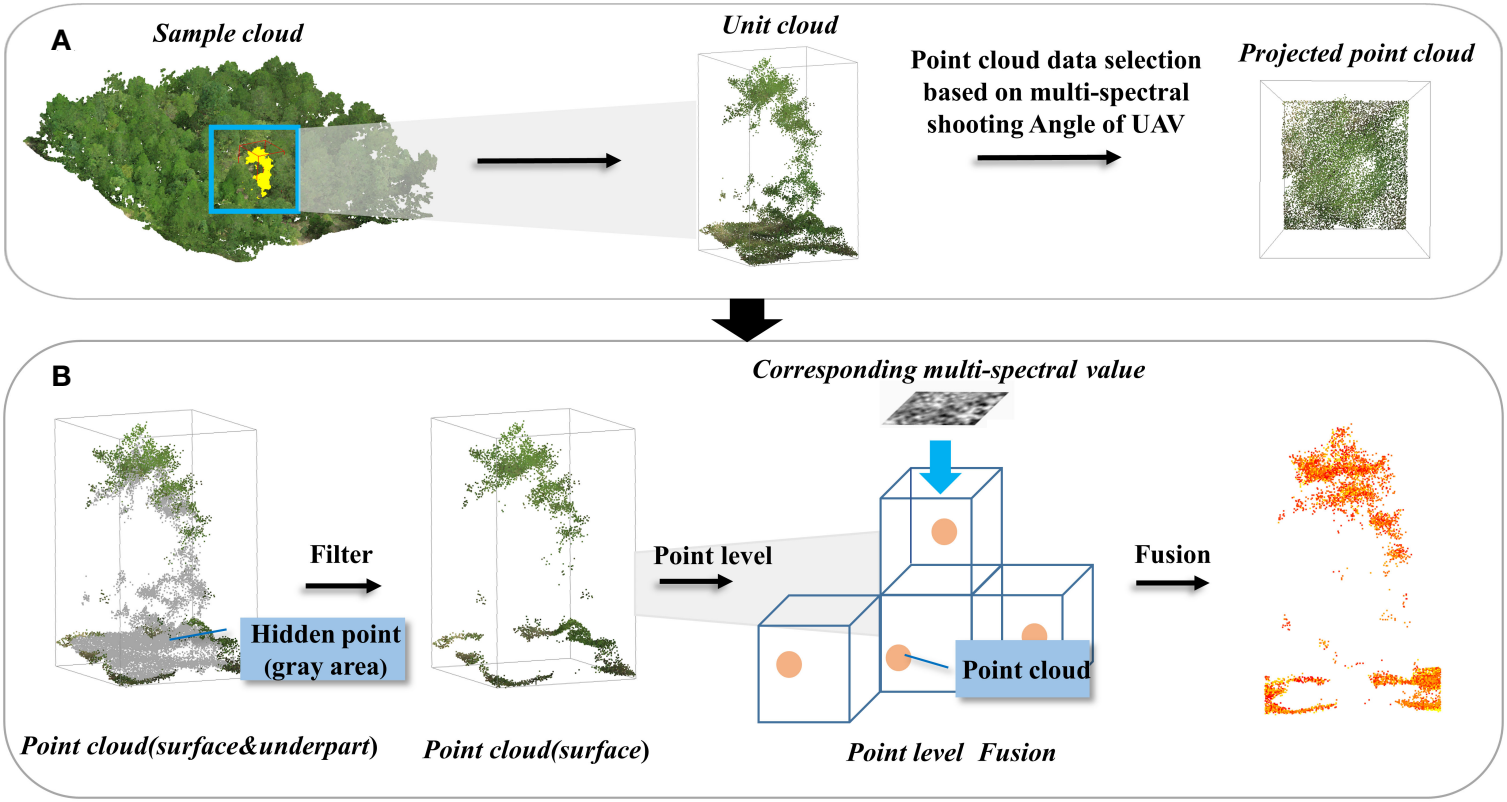
Schematic of multispectral image fusion to the point cloud, **(A)** angle determination, **(B)** hidden point removal and data fusion.

**Figure 4:**
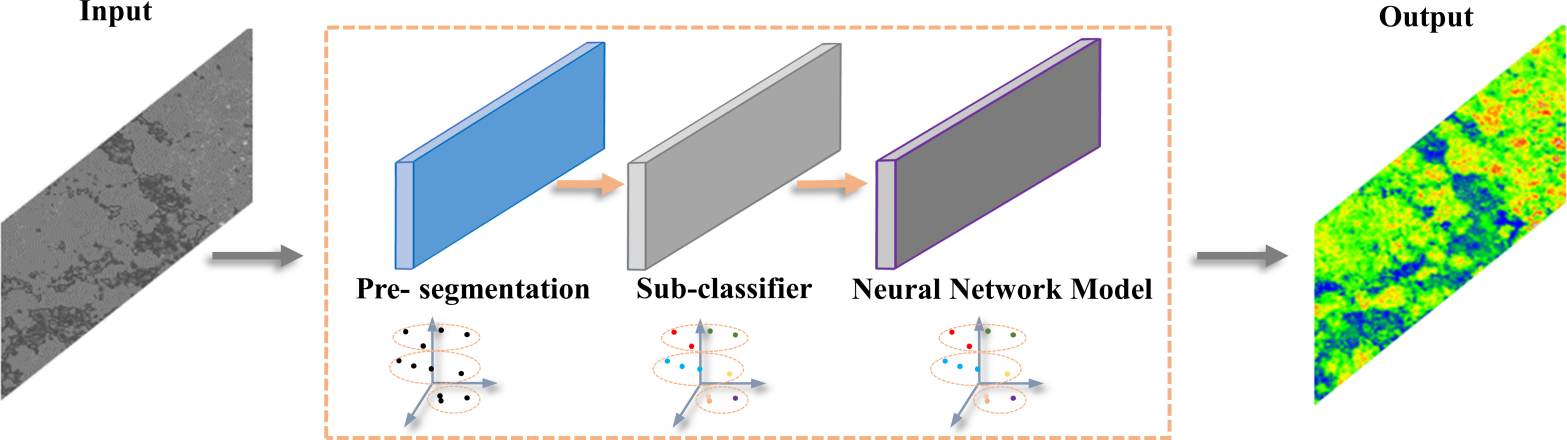
Schematic of the inversion model for multispectral values of the point cloud.

After feature fusion, the point cloud dataset was still quite massive and had a modest, unpredictable spectral distribution. To enhance a potential model’s capacity to detect features, it was essential to highlight the spectral distinctions between various kinds of features in the dataset’s jumbled information dispersion structure. In this study, firstly, the outlier detection method of the isolated forest (Fei et al., 2008) was used to remove the outliers from the seven-dimensional point cloud dataset to reduce the redundancy of the subsequent model training data (**Figure 3**), then, based on the removal of the outliers, the K-means algorithm(Hartigan and Wong, 1979) combined with the elbow method was used to determine the number of clusters to amplify the spectral distribution features within the point cloud (**Figure 4**).

1)Isolated Forests Algorithm: Aiming at the outliers, noise, and deviation data existing in the massive high-dimensional point cloud data set in this study, the isolated forests algorithm was used to remove the outliers. The isolated forests algorithm is an unsupervised outlier detection method, which uses the number of times required by random cutting isolated data sets to describe the anomaly degree. Compared with traditional anomaly detection methods, this algorithm does not need to normalize sample data and has stronger generalization performance, can be well applied to high-dimension data sets.

The main algorithm ideas of isolated forests are as follows:

a) Randomly sample the data to be detected, select specific points as sub-samples to construct an isolated tree, and input the samples into the forest.

b) Test each isolated tree, calculate the test data on the tree along the corresponding conditional branch until it reaches the leaf node, and record the path length h(x) in the process.

c) Calculate outliers of samples at leaf nodes, calculate outliers though (3) and (4):


(3) $$s(x,\psi )=2^{-\frac{E(h(x))}{c(\psi )}}$$

(4) $$c(\psi )=\{\begin{array}{cc} 2H(\psi -1)-2(\psi -1)/\psi & \psi >2\\ 1, & \psi =2\\ 0, & otherwise \end{array}$$

d) Repeat the above steps to continuously divide the binary tree into leaf nodes.

Where S represents the abnormal fraction, C represents the average path length of the binary search tree, h(x) represents the height of x in each tree, H represents the harmonic number, and C is used to normalize the calculation results. The larger the calculated path length C is, the smaller the abnormal fraction S is. If S is close to 1, it is judged as an anomaly, if S is much less than 0.5, it is judged as a non-anomaly.

2)K-means Clustering Algorithm: Due to the obscure spectral distribution pattern inside the spectral-fused point cloud dataset and the disturbance of the enormous sample size, the spectral-spatial feature dispersion appears tough to integrate. Consequently, the K-means method was selected for the primary classifier in our work, which groups the spectral attributes within each dataset to again strengthen feature distribution consolidation and facilitate object recognition in subsequent models. The followings are the basic clustering principle:

- Randomly initialize K clustering center points, calculate the distance between each point in the data and K clustering center points,
- Assign each data point to the cluster center nearest to the cluster center,
- Recalculate clustering centers for each category,
- Repeat steps 2 and 3. When the set number of iterations is reached or the mean vector of the cluster class is no longer changed, the model construction is completed and the results of the clustering algorithm are output.

The Euclidean distance between the data object and the clustering center in the spatial data is calculated though (5), where X is the data object, C_i_ is the i-th clustering center, m is the dimension of the data object, and X_j_ and C_ij_ are the attribute values of X and C_i_, respectively.


(5) $$d(X,C_{i})=\sqrt{\sum_{j=1}^{m}{\left(X_{j}-C_{ij}\right)}^{2}}$$

The elbow strategy was used in this work to identify the last number of clustering to guarantee that the degree of grouping satisfies the requirement of feature statistical distribution of the point cloud data set. By drawing the distribution diagram of the sum of squares of error (SSE) and a number of clusters (K) and finding the inflection point of the curve distribution, the optimal number of clusters’ K value can be determined. SSE value represents the square of the distance between the points of each cluster and their centroid.

K=8 was discovered to be the optimum K value for feature grouping in this dataset. After determining the clustering centers and boundaries of the object classes, a pseudo label was assigned to each point cloud internal feature spectral value in ascending order from 1 to 8, each pseudo label represented a type of genuine spectrum, completed the NIR dimensionality reduction, and was then used for the following feature band mapping lookup. The elbow method is formulated as (6), where C_i_ is the clustering center, p is the sample point in C_i_, and m_i_ is the centroid of C_i_:


(6) $$\text{SSE}=\sum_{i=1}^{k}\sum_{p\in C_{i}}/p-m_{i}{/}^{2}$$

#### 2.3.3 Model construction

1) Datasets: On the premise of ensuring the correct fusion results, the data enhancement processing of outlier removal and feature re-clustering was carried out for the fusion point cloud data set. Since the inner spatial patterns of the spatial-spectral characteristics of the point clouds were not yet fairly obvious, our study performed a variable correlation analysis on the enhanced point cloud set and chose the Spearman correlation coefficient (7) to depict the variable correlation within the differential set of points and to investigate the impact that of various qualities of the point clouds on the feature inversion effects.


(7) $$\rho =1-\frac{6\sum_{i=1}^{n}{\left(x_{i}-y_{i}\right)}^{2}}{n(n^{2}-1)}$$

where n is the sample size, x is the independent variable, y is the dependent variable, and ρ is the correlation coefficient, where values closer to 1 indicate a higher correlation between variables.

The point clouds and spectra of three different types of surface objects from sample sets were combined in this study. Three types of typical ground objects (bare ground, herbs, and canopy) were taken during the annotation process and manually annotated in Cloud Compare in las format to explore the spectral distribution norms of various ground items. Each point cloud’s properties were recorded as (X, Y, Z, R, G, B, NIR), where (X, Y, Z) referred to the point cloud’s initial coordinates, (R, G, B) referred to its true color data, and NIR referred to its fusion pixel value. The aberrant point clouds were removed using the isolated forest algorithm, the secondary classification was completed based on the corresponding label applied to each type of point cloud in line with the NIR distribution in the clumping of category points in various locations as well as the above K-means clustering algorithm. Since the distribution of the (X, Y, Z) values had magnitude differences, the coordinates were normalized to create a data set with a normal distribution by dividing by the standard deviation after subtracting the mean value, and (R, G, B) were normalized to be between 0 and 1 to unify the magnitude, the point cloud was then stored in the format: (x, y, z, r, g, b, label), (x, y, z) was the point cloud coordinates after standardization, (r, g, b) was the true color value after normalization, and label corresponded to the real value of NIR dimension reduction for each point cloud. Finally, the processed data set was then split into a training set, test set, and verification set in an 8:1:1 ratio and delivered into the DL model.

2) Training Settings: The 3D point cloud segmentation task groups the same class of points into a subset based on the semantic information of a given point cloud, compared to 2D semantic segmentation, 3D semantic segmentation can distinguish spatial objects in greater detail, semantic segmentation methods based directly on point clouds can perform the segmentation task effectively without losing structural information (Bello et al., 2020). In hopes of comprehending the internal knowledge of the fused point cloud dataset, 5 distinct DL models, including PointNet, PointNet++, PointMLP, Point Transformer, and Point Conv, were employed in this study, all of the above methods directly used 3D point cloud as input. To ensure consistency of comparison results, the parameters of the 5 different models were all set to learning rate = 0.05, batch size = 4, and epochs = 150. The specific operating environment and configuration of this study are shown in **Table 2**. The main libraries used by the five types of deep learning algorithms include matplotlib, numpy, scikit-learn, and h5py, among others.

**Table 2:** Environments and versions.

Name	Parameters and versions
CPU	Intel Xeon™ E5-2680 v4 @2.4 GHz
GPU	NVIDIA GeForce RTX 2080Ti (11 GB)
RAM	16 GB
OS	Windows 10 Professional
ENVS	PyTocrh 1.10.0 + Python 3.7

Two indexes in semantic segmentation were adopted as accuracy evaluation indexes to objectively assess the accuracy of point cloud semantic prediction: overall accuracy (oAcc) and mean intersection ratio (mIoU). In this work, point cloud NIR prediction was regarded as a part segmentation task, the NIR value functioned as the corresponding label required for segmentation. The ratio of positive case prediction to positive case prediction is known as oAcc. The intersection and union ratio between true and anticipated values in various categories. The formulae for the evaluation indexes are as (8) and (9):


(8) mIou=1k+1∑i=0kpii∑j=0k pij+∑j=0kpji−pii

(9) $$oAcc=\sum_{i=0}^{K}\frac{p_{ii}}{\sum_{j=0}^{K}p_{ij}}$$

where i represents the real value, j represents the predicted value, p_ij_ represents the prediction of i as j, and k is the total category.

By drawing the distribution diagram of the sum of squares of error (SSE) and a number of clusters (K) and finding the inflection point of the curve distribution, the optimal number of clusters’ K value can be determined. SSE value represents the square of the distance between the points of each cluster and their centroid.

## 3 Results

### 3.1 Visual analysis of fusion results

**Figure 5:**
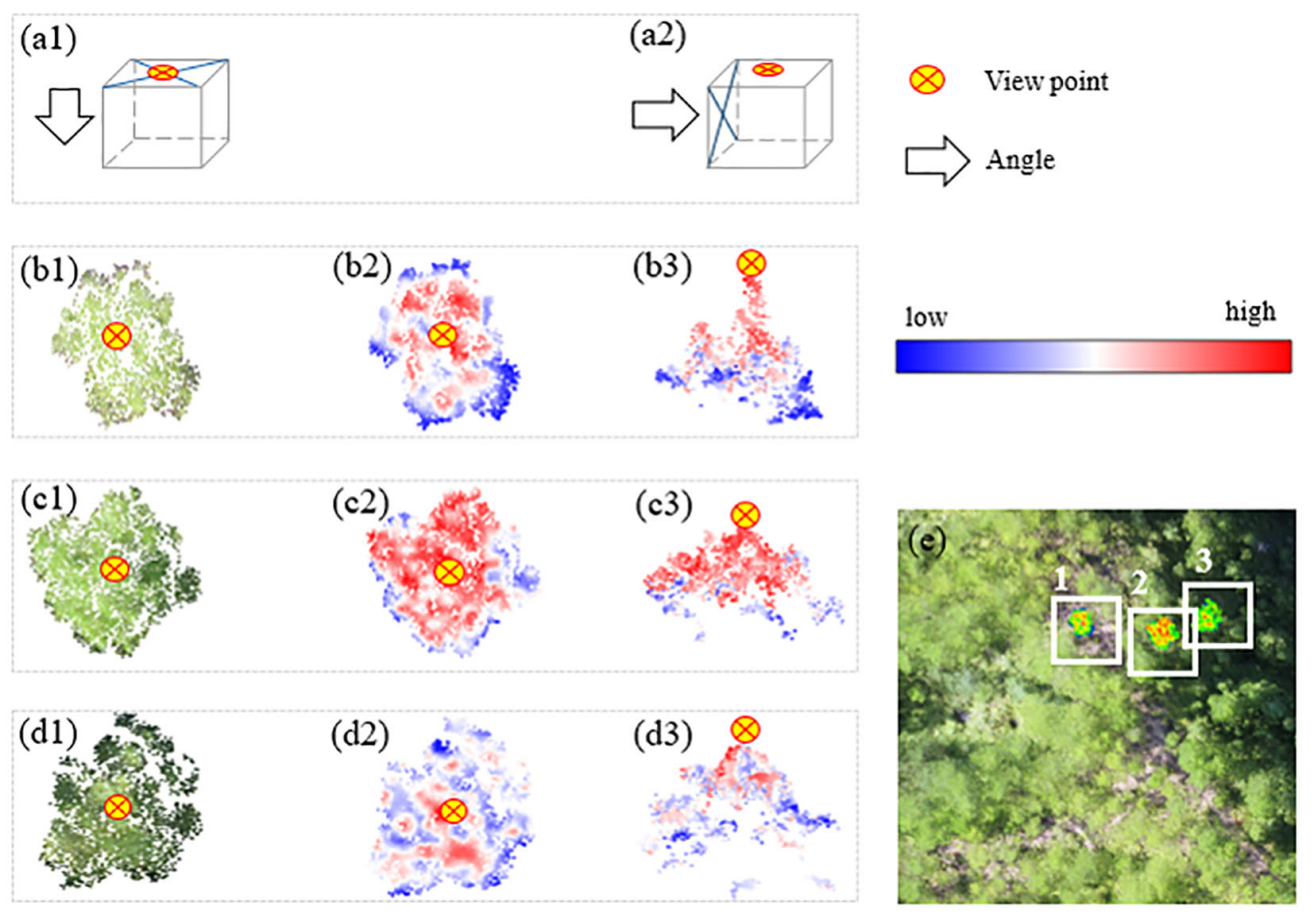
Schematic of the effect of space-spectrum fusion. (a1-a2) Top view and side view. True color of (b1) sample wood 1, (c1) sample wood 2, and (d1) sample wood 3. NIR distribution overlooking (b2) sample wood 1, (c2) sample wood 2, and (d2) sample wood 3. NIR distribution for the side view of (b3) sample wood 1, (c3) sample wood 2, and (d3) sample wood 3. (e) Actual distribution of sample wood 1-3.

**Figure 6:**
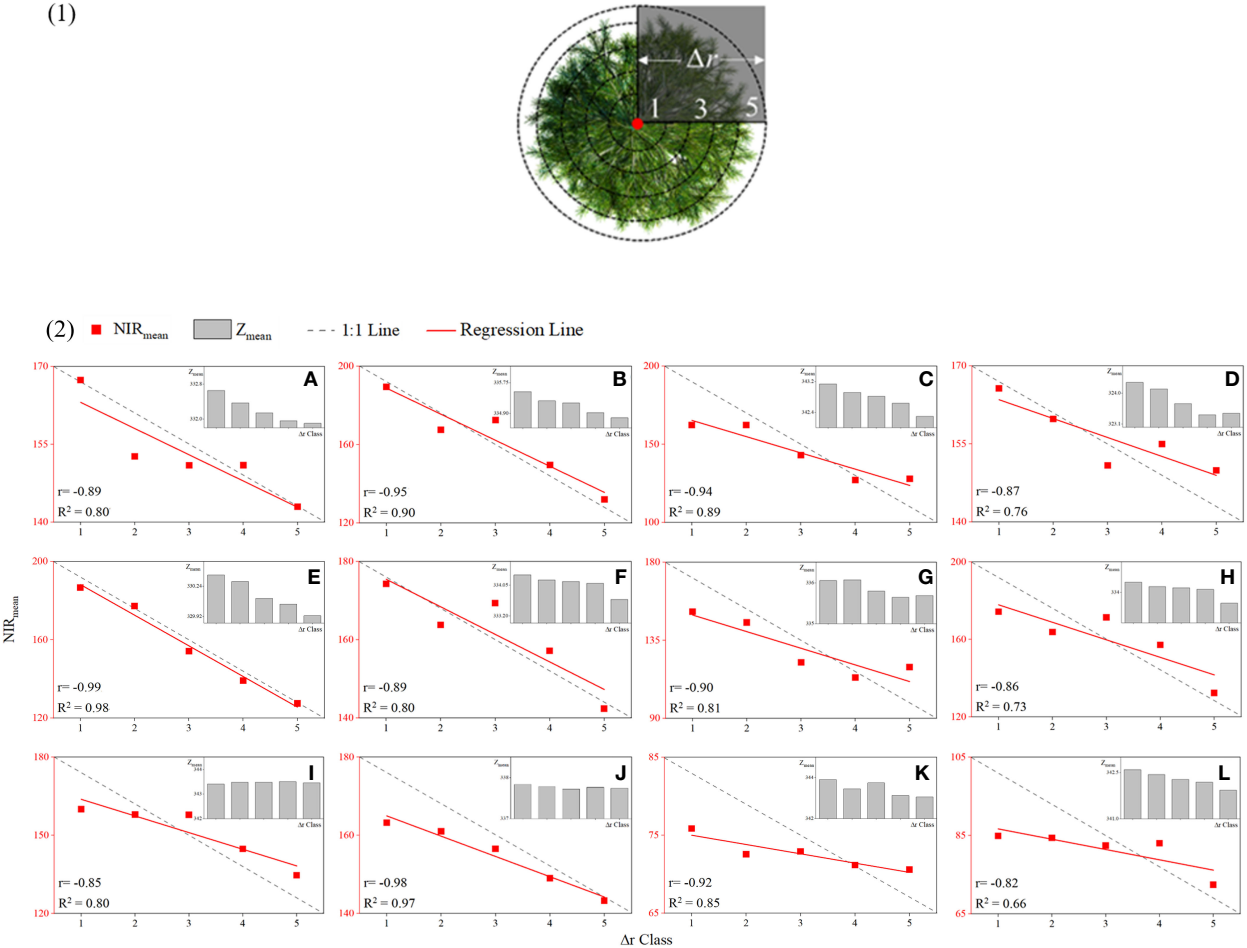
Scatter diagram of the spatial distribution of NIR values. (1) Classification of canopy point clouds, (2) scatter diagram of the spatial distribution of NIR values of random individual trees: **(A-L)** sample trees 1–12.

The point cloud data and the spectral data of the same position were matched using the hidden point removal method and the spatial collinear equation. The aerial photography angle of the UAV was taken to remove hidden points, and a single point cloud was maintained in the Z-value channel within a specific unit for multispectral assignment after the non-fusion point cloud was eliminated according to this angle. Three sample trees from sample 3 were selected at random to illustrate the distribution of their NIR after fusion, as displayed in (**Figure 5**). Where the color is red, the NIR value is higher, where the color is dark blue, the NIR value is lower. Three trees’ NIR values were radially spread from the center of the crown portion in the overlooking view, hitting a maximum point before gradually decreasing as the radius increased. Since the height difference between sample-woods 1 and 2 was greater than that between sample-woods 3, the NIR value peaked in the side view at the crown vertex, while the distribution difference of NIR on the vertical section was more significant. The tree apex was set as the center of the circle, the tree crown was divided into 5 levels with equal distances, and 12 sample trees were arbitrarily assigned from the sample sets (**Figure 6** **-1**). The average elevation value and average NIR distribution of point clouds in each level were recorded as Z_mean_ and NIR_mean_, and the scatter diagram of NIR value and canopy grade distribution of single tree point clouds was drawn (**Figure 6**–**2**). The Z value and NIR value of the six sample trees all demonstrated a declining trend from the tree apex. The NIR value and radius level R^2^ were the lowest and highest, at 0.66 and 0.98, respectively. These results indicated that the spatial distribution of NIR correlates positively with the height of a single tree and that it gradually rose with an increase in the Z value of a single tree point cloud, peaking at the apex of the tree. Typically, the NIR value at the canopy section’s center tended to be greater than that at the edge.

### 3.2 Quantitative analysis of parameters correlation

**Figure 7:**
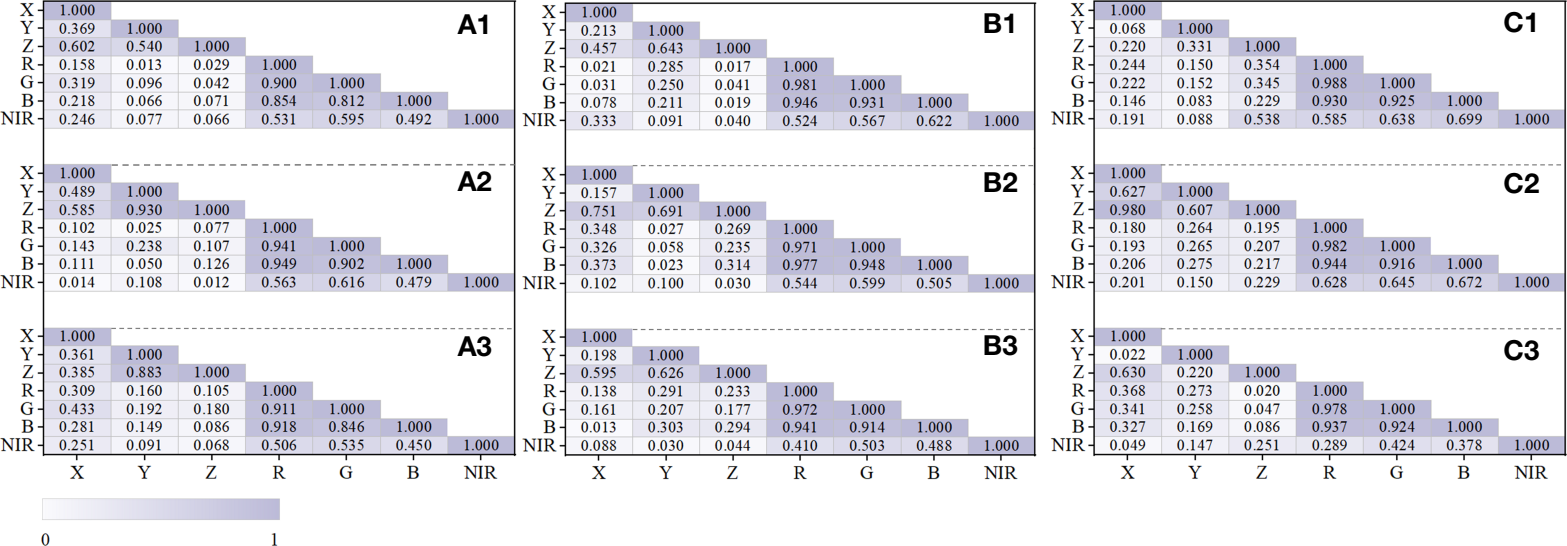
Correlation analysis of point cloud features in samples. The canopy point cloud correlation of (a1) sample 1, (b1) sample 2, and (c1) sample 3. The bare ground point cloud correlation of (a2) sample 1, (b2) sample 2, and (c2) sample 3. The herb point cloud correlation of (a3) sample 1, (b3) sample 2, and (c3) sample 3.

Our study analyzed the internal attribute correlation of point cloud variables via an internal attribute correlation analysis on the fused point cloud dataset that has been separated into geographical types utilizing the Spearman coefficient to evaluate pairwise correlations among variables. All attributes showed substantial correlation at the significance level P<0.01, according to the inner correlation analysis of 830,814 point cloud data (X, Y, Z, R, G, B, NIR) from the three samples (**Figure 7**). The correlation distribution of the sample set attributes revealed several characteristics: the spatial variable set (X, Y, Z) had a strong self-correlation inclination, with the bare class’s Z-Y in sample set 3 having the greatest correlation and the herb class’s Z–Y having the least correlation (ρ_spatial_min_ = 0.022, ρ_spatial_max_ = 0.980). In comparison to X and Y and Z and X, there was a stronger link between Z and Y. The correlation between the three primary colors was weakest for the crown class in sample set 1 and finest for the canopy class in sample set 3(ρ_optical_min_ = 0.812, ρ_optical_max_ = 0.988) in the spectral variable set (R, G, B, NIR), which likewise displayed a significant self-correlation trend. NIR and the three primary colors had a less significant correlation than the three primary colors themselves. The R-NIR of the herb type in sample 3 had the smallest connection, while the B-NIR of the canopy type in sample 3 had the strongest link (ρ_nir_min_ = 0.251, ρ_nir_max_ = 0.699). The self-correlation with the three primary colors ranged from 0.45 to 0.65. In terms of the correlation distribution of ground objects, the spatial property of bare land had the strongest correlation, and the highest was X to Z (ρ_land_spatial_ = 0.980) in sample 3, followed by the canopy, and the lowest was the herb. This could be due to the bare ground allocation failing to display a staggered cloud of highs and lows, in contrast to the herbaceous and canopy layers. As a result, the differences were less pronounced on the vertical scale, with Z values fluctuating gradually and cooperatively with X and Y values. Compared with vegetation and canopy, bare soil had a spectral attribute correlation higher than 0.9, and the gap in actual reflectance between bare soil and the other two categories of surface items was consistent with this result.

### 3.3 Quantitative analysis of models accuracy

In this study, the test set and validation set data of point cloud data fused with three different depression closures were tested on top of five models, and the accuracy of the results is shown in **Tables 3**–**5**. The data revealed that, in the experimental results of sample 1, PointMLP performed well on all categories of ground objects, with the canopy category establishing a maximum accuracy of 0.84, and PointNet++ followed behind. The performance of PointMLP was also the best in sample 2, and its precision for the bare ground class was the most significant at 0.84. The performance of PointNet++ ranked second. In sample 3, PointMLP stood out for data of bare ground and herbs. The bare ground had the optimum precision in samples 2 and 3, in sample 1, while canopy had the highest accuracy, and the three samples’ estimates of the effect of herbs fell between bare soil and the canopy.

**Table 3:** Sample 1 performance evaluation of various algorithms.

Model	Tree		Land		Grass	
	mIoU	oAcc	mIoU	oAcc	mIoU	oAcc
PointNet	0.68	0.78	0.54	0.63	0.47	0.62
PointNet++	0.70	0.82	0.65	0.79	0.49	0.63
PointMLP	0.72	0.84	0.65	0.79	0.50	0.66
Point Transformer	0.76	0.79	0.64	0.70	0.58	0.68
Point Conv	0.68	0.79	0.60	0.75	0.48	0.64

**Table 4:** Sample 2 performance evaluation of various algorithms.

Model	Tree		Land		Grass	
	mIoU	oAcc	mIoU	oAcc	mIoU	oAcc
PointNet	0.46	0.53	0.69	0.73	0.52	0.60
PointNet++	0.52	0.67	0.71	0.83	0.54	0.69
PointMLP	0.55	0.70	0.75	0.84	0.59	0.72
Point Transformer	0.59	0.63	0.58	0.65	0.55	0.63
Point Conv	0.40	0.67	0.63	0.76	0.48	0.56

**Table 5:** Sample 3 performance evaluation of various algorithms.

Model	Tree		Land		Grass	
	mIoU	oAcc	mIoU	oAcc	mIoU	oAcc
PointNet	0.59	0.65	0.57	0.62	0.45	0.64
PointNet++	0.64	0.77	0.63	0.76	0.62	0.76
PointMLP	0.66	0.77	0.65	0.78	0.64	0.78
Point Transformer	0.46	0.63	0.42	0.67	0.68	0.73
Point Conv	0.46	0.55	0.52	0.66	0.46	0.63

Considering the attribute differentiation of the correlation degree of point cloud variables of different land classes, the optimal prediction effect of the bare land class dataset might be because spectral variables deepen the cognizable range of the DL model to a certain extent. The herb set and canopy set had moderate spectral correlations, however, since the information richness of the spatial features in the canopy set was greater than that in the herb set, the canopy set had a marginally stronger predictive ability than the herb set.

**Figure 8:**
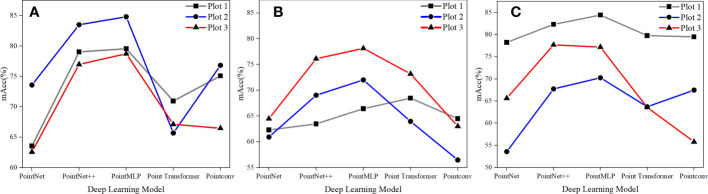
Comparison of deep learning models for three types of sample sets. **(A)** land, **(B)** grass, and **(C)** canopy.

**Figure 8** utilizes a point-line chart to evaluate the DL model’s performance in sample sets with various canopy thicknesses. Sample sets 1, 2, and 3 are denoted by the broken lines in gray, blue, and red, and the actual canopy thicknesses corresponding to each sample set are 0.20, 0.35, and 0.60, respectively. As the canopy density of the sample set grew, there was no significant apparent difference in the NIR prediction outcomes for various point clouds. As evidenced in (**Figure 8**), the three sample sets’ accuracy ranged around 0.45, with 0.65 to 0.85 being the greatest. It’s possible that, during the processing of the data set, sampling was performed to minimize the gap magnitude between the point clouds of various ground objects.

As a byproduct, the distribution of ground point clouds in samples with different canopy densities was fairly uniform, and the number of canopy points did not reduce as canopy density rose. As a result, while canopy density increased, the amount of bare soil and herb points did not. Based on the results of the model selection, PointMLP had the best performance among the five model algorithms, followed by PointNet++, and the other three models performed intermediate, with precision ranging between 0.40 and 0.70. This might be because massively complicated data sets restrict the ability to employ sophisticated and thorough local geometric feature extraction techniques, including convolution, graph, and attention mechanisms. The network architecture adopted by PointMLP is residue feed-forward neural MLP, and its local geometric affine module may adjust to transforming local region point characteristics. Furthermore, the model could integrate residual linkages to create depth features, which is more appropriate for the overwhelming amount of information contained in the fusion point cloud dataset used in this work.

### 3.4 Quantitative analysis of spectral reconstruction results

**Figure 9:**
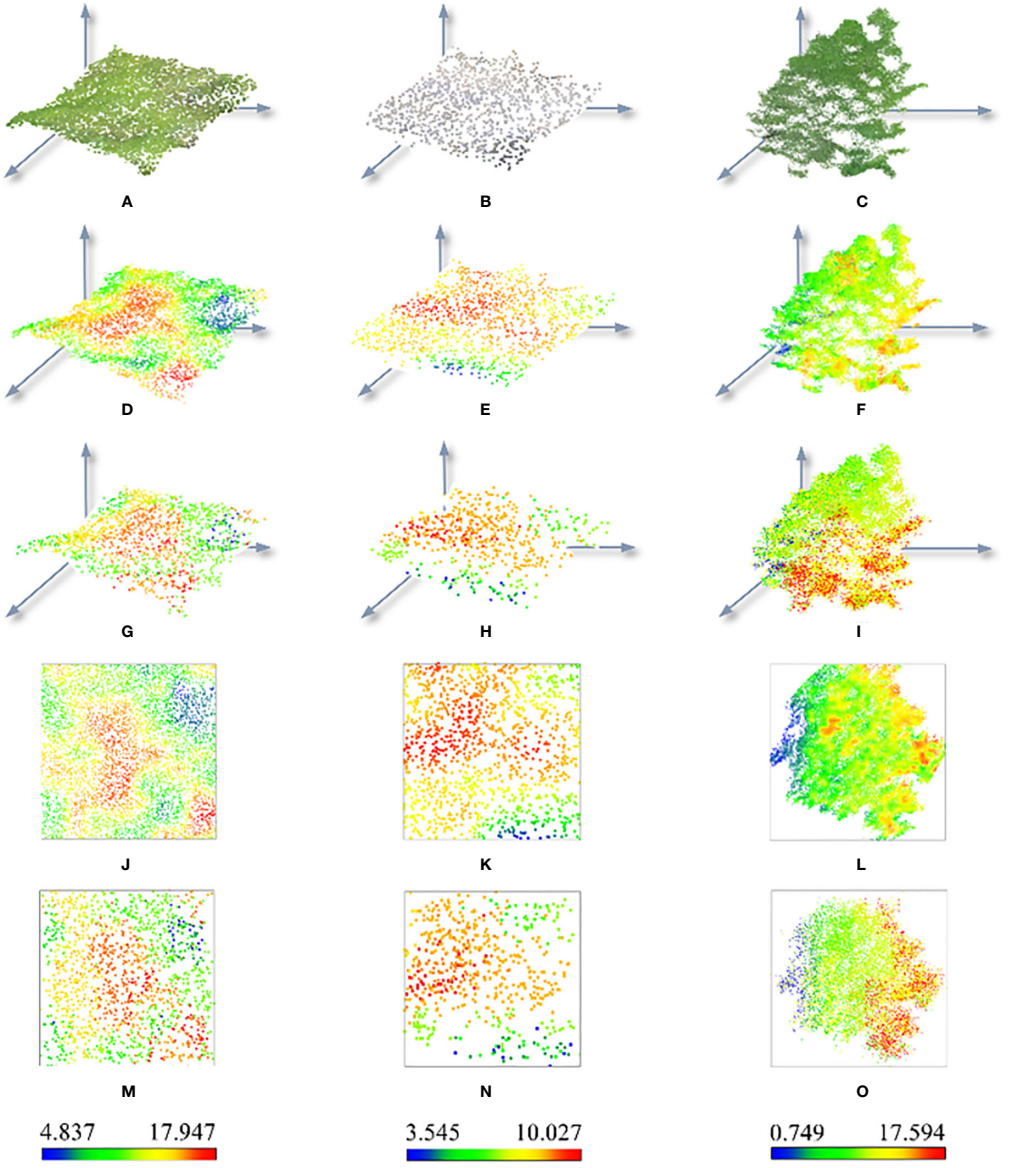
Distribution of predicted and true values of NIR grassland, bare soil, and canopy for three types of sample set. RGB format of **(A)** grassland, **(B)** bare soil, and **(C)** canopy. 3D distribution of true values of NIR of **(D)** grassland, **(E)** bare soil, and **(F)** canopy. 3D distribution of NIR predicted value of **(G)** grassland, **(H)** bare soil, and **(I)** canopy. Platform of NIR real values of **(J)** grassland, **(K)** bare soil, and **(L)** canopy. Platform of NIR predicted values of **(M)** grassland, **(N)** bare soil, and **(O)** canopy.

Point clouds outside the sample 1 training set were selected for the spectral reconstruction of all three types of ground objects. The reconstruction effect was assessed and scrutinized in conjunction with the frequency of the spectral values. The NIR values in the areas from blue to red steadily increased in the NIR prediction distribution diagram of the point cloud (**Figure 9**). Though the overall distribution situation was essentially consistent with that before inversion and the spatial distribution characteristics of the three types of ground objects resembled each other after inversion, there still exist several inadequacies in the details. The inversion values lost more border details and the NIR values were exaggerated at higher levels, but the genuine NIR values of grassland samples were expressed in more detail at the edges. The NIR distribution of the bare soil samples before and after inversion was consistent with the azimuth, but the information output at the boundary was also slightly lacking, and the true value of NIR tended to be lost. The overall distribution of canopy samples before and after inversion was similar, but the local overestimation was more prominent, and the NIR at the canopy boundary was higher.

**Figure 10:**
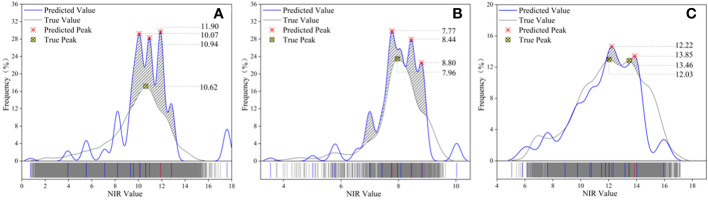
Comparison of distribution frequencies of predicted and true NIR values in three types of sample sets. **(A)** canopy, **(B)** bare land, and **(C)** grassland. The blue curve represents the predicted value, the gray curve represents the true value, the red symbol represents the predicted peak value, the yellow symbol represents the true peak value, and the diagonal line represents the relative coincidence of the wave crest.

**Figure 11:**
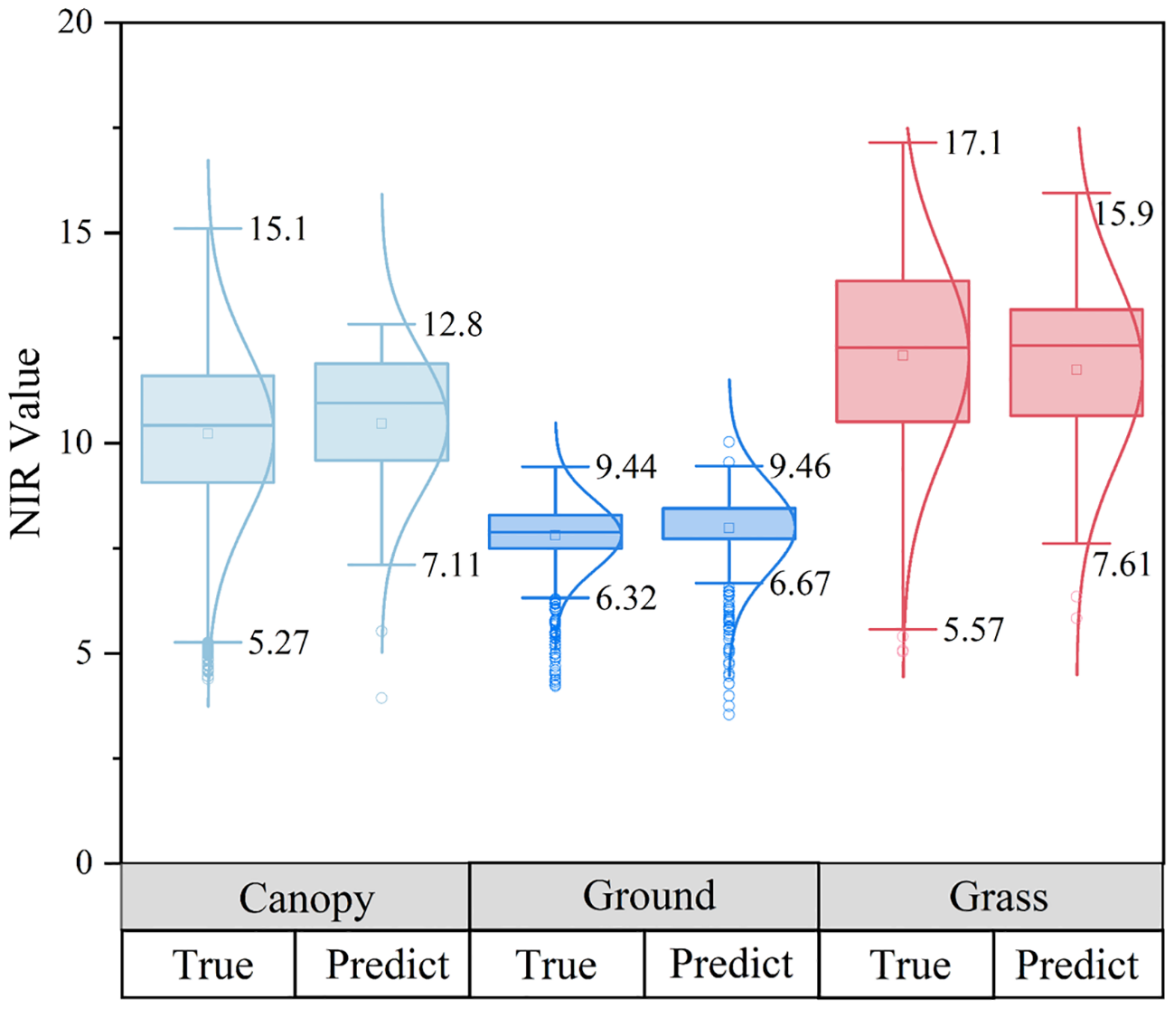
Comparison of the true distribution of predicted values of three types of sample sets.

Three different ground objects were used to draw the NIR distribution frequency curve, the blue curve represents the inversion result of the DL network, and the gray curve reflects the true value. **Figure 10** represents the canopy, bare soil, and grassland in succession. The predicted value contained three peaks and was scattered in the range 10.07 to 11.90, whereas the true value of the canopy sample only had one peak (NIR_max_ = 10.62), and the two peaks had converged, as seen by the maximum difference of 1.27 between the anticipated value and the actual value. The predicted value for the bare ground sample set comprised three peaks (7.77-8.80), however, the actual value had just one peak (NIR_max_ = 7.96), between the true worth and the forecasted value, and the maximum difference was 0.83, however, the two peaks’ redistributive spans generally followed the curve’s general tendency. The grassland samples had two peaks, with the real peaks at 12.03 and 13.46 and the estimated peaks at 12.22 and 13.85, and the maximum gap between the predicted value and the real peak was 0.39. The median values of the predicted values of the canopy and bare land were all higher than the true values, as determined by a contrast of the allocation between the estimated parameters and the real values of the three samples (**Figure 11**), while there was little difference in the grass class. The model’s predictive outcomes and the actual values both manifested the traits of a normal distribution.

The prediction values replicated the initial sample distributed across the deviant range of data points, indicating that identification recognition for all attributes in the point cloud dataset was well performed by the model. The distribution curves of the three types of samples showed local oscillation and deviation estimation, among which overestimation accounted for a large portion, as well as characteristics of feature imbalance and detail loss. Even so, the NIR distribution frequency graphs of all sample types exhibited positive consistency within the group. The model may meet the requirements of global inversion because the global distribution of the NIR forecast was essentially compatible with the true value.

## 4 Discussion

### 4.1 Spectral-spatial distribution of the forest after the fusion of multispectral images and point clouds

In our study, a stand’s multispectral values were assigned to a point cloud based on spatial orientation, revealing a correlation between the spectral distribution of stand and the spatial location on the point cloud. Prior research has demonstrated that multi-spectrum and LiDAR point cloud-based spatial data fusion can significantly increase the precision of ground-object segmentation and classification (Kai et al., 2022). The fusion approach revealed a substantial relationship between the NIR value of a stand and (RGB) spectral attribute value (P< 0.01). Based on the specific spectral distribution of individual trees at the point cloud scale, the 3D spatial distribution features of the stand point cloud attributes possessed a pronounced self-correlation, and the spectral distribution had some spatial regularity. According to previous studies (Morsy et al., 2016; Eva et al., 2021), the spectral distribution pattern of various tree species and ground classes was anisotropic. The internal self-correlation (ρ = 0.988) of the spectrum variables (R, G, B, and NIR) and spatial variables (X, Y, and Z) were both considerably higher than the correlation between the two variable types (ρ = 0.433); in comparison with those of the canopy and herb layer, the bare ground had a relatively high spatial correlation and spectral correlation. The decay of radioactivity from the tree apex to the tree crown, which has a significant relationship (*R* ^2^_max_ = 0.9842) with the point cloud Z value, represents the NIR diffusion of the canopy. There was no significant association between the two when the canopy was sparse. When the canopy distribution was uniform, the NIR value at the center of the canopy was generally higher than that at the crown edge. Therefore, rather than neglecting the stand condition for unified measurement, studies of the inversion of stand-related metrics based on NIR values retrieved from 2D images should consider the canopy density.

### 4.2 New method for stand spectral inversion

In this study, a feature enhancement method was developed for isolated forests combining K-means clustering with the DL algorithm. The results of this study suggest that the fusion of a high-resolution remote sensing image and a point cloud is ideal, and that this feature augmentation strategy is capable of improving the degree to which model details can be detected (Shang et al., 2023). To illustrate the advantages of the method, DL model performance and forest spectral inversion results were evaluated, as discussed below.

The point cloud DL method can separate components, classify point clouds of various shapes, and learn the internal rules of a dataset (Siddiqui and Hyunsik, 2022). Point convergence with complicated attribute features is better suited for the model directly employing the pure MLP network architecture (oAcc_canopy_ = 84.40%, oAcc_land_ = 84.81%, oAcc_grass_ =78.13%), consistent with previous results (Xue, 2022), and other segmentation techniques perform marginally poorer. The MLP network framework can implement end-to-end data processing and retrieve the intricate details of point convergence for end-to-end semantic segmentation of fused point cloud datasets containing high-dimensional information. We therefore chose the PointMLP algorithm as our basic DL method in combination with feature enhancement for stand spectral inversion.

The spectral distribution in forests is vital for monitoring, as different ground objects have distinguishable spectral distribution properties (Feng Q. et al., 2022). However, studies of the spectral distribution of the canopy, bare ground, and grass are lacking. On the basis of establishing feature-enhanced data sets, semantic segmentation and prediction were performed on the opposite end of three different types of ground object point clouds in this study by integrating the point cloud-spectrum fusion algorithm with the DL model with the general MLP architecture. The key findings of the quantitative examination of inversion values show that the difference between real values for the three types of sample sets and predicted values were generally consistent (NIR-Difference_max_ = 1.27); the model displays accurate inversion outcomes as well as the outstanding capacity to recognize global features. As shown in **Figure 10**, the three different types of sample peak intervals on the normal curve of the NIR frequency distribution before and after inversion were generally uniform, demonstrating that the inversion results are in line with the real NIR sample distribution. The NIR values of different land classes possess convergent distributions. Ground object clustering inversion performances were better near the center than at the boundary, and the dispersion traits of outliers in the max sample set were also well preserved. Although the model has an excellent degree of dependability and can accommodate global inversion, there are some cases of locally uniformly inflated results, and its resolution is inadequate.

### 4.3 Future improvements

Three aspects of forestry remote sensing work may benefit from our research. First, the proposed forest point cloud and multispectral fusion methods can provide technical support for image element and point cloud fusion in subsequent studies. Second, when forest spectral values showed a unique distribution, NIR values were generally higher in the center of the canopy than at the edge of the canopy. These results indicate that in the study of forest stand attributes based on 2D image inversion, forest conditions should not be measured uniformly but should consider the distribution of forest canopy densities. Third, forest spectral detection is a basis for forest management (Bolin et al., 2022). It is difficult to obtain true 3D spectral information for forests with restricted spectral values, creating a key gap in knowledge; the 3D spectral inversion technique proposed in this study breaks resolves this issue to a certain extent and can provide an important reference for relevant forest departments.

Nevertheless, there are two main sources of uncertainty in our research. First, during the forest data collection process using the UAV on-board multispectral camera or LIDAR sensors, coordinate uncertainty in spatial point fusion can result from self-systematic errors (Zha et al., 2023). Aimed at this problem, we used high-precision GPS to locate the coordinates of the four points in the sample plots to ensure the accuracy of the coordinates. Second, in the fusion of 3D point cloud data with 2D multispectral data, there is an impact of large amounts of redundant point cloud data beyond the surface of land features; accordingly, we conducted hidden point removal to minimize this issue.

Several unresolved issues remain. First, due to the inadequacy of prior knowledge in the inversion process, only the method of retrieving spectral values with the spatial structure of the point cloud was tested, and the inner mechanism relating spatial and spectral information remained unclear. Second, data sources were dimensionally limited; we only obtained 2D multispectral data. Further studies of multi-angle multispectral data may be helpful (Wang R. et al., 2021; Yuan et al., 2021), indicating that more information on the distribution of spectral values in the middle and lower parts of canopy would be learned by the model. Moreover, the spectral class was limited, fusion and inversion with hyperspectral images will provide richer spectral information (Xu, 2023), thereby extending the 3D spectral breadth of forests in terms of species.

## 5 Conclusion

This study set out to determine a method for inverting 3D spectra of *Pinus massoniana* stands based on multispectral imagery and point cloud data; spectral information and 3D structure information for the stand were collected. The most applicable method was PointMLP, the highest oAcc was 0.84, the highest mIoU was 0.75, the peak distribution interval of the prediction curve tended to be consistent with the true value, and the maximum difference between the predicted value and the true value of the point cloud spectrum was 0.83. These results reveal that the point cloud spectrum-spatial fusion method with combined hidden point removal can effectively replicate the 3D spectral distribution of the stand, with broad implications for the prediction of spectral attributes and precise global stand spectral inversion. In addition, compared with the 3D spectral inversion of stands at the present stage, this work based on the removal of redundant point cloud points, reducing their impact on point cloud fusion work, represents the first attempt to invert spectral values in point-to-point form. The study contributes to the precise extraction of stand attribute information from a point cloud and addresses the limitations of 2D forest spectral information to a certain extent. A limitation of this study is that the embeddability module based on the traits of datasets was not considered, leveraging a complete deep-learning framework for model comparison. Future research should investigate the spectral distribution concepts related to forest ground features probed with the aid of broader fusion datasets and account for complex situations, such as phenology, pests, and illnesses.

## Data availability statement

The raw data supporting the conclusions of this article will be made available by the authors, without undue reservation.

## Author contributions

Conceptualization: SL, LX, and WF; methodology: SL, LX, and WF; software: SL, LX, and WF; validation: ZS and WJ; formal analysis: ZS and WJ; investigation: ZY and WF; resources: ZY; data curation: SL, LX, and WF; writing—original draft preparation: SL, LX, and WF; writing—review and editing: SL, LX, and WF; visualization: SL, LX, and WF; supervision: WF; project administration: WF; funding acquisition: WF. All authors contributed to the article and approved the submitted version.
