# Corrigendum: Forest stand spectrum reconstruction using spectrum spatial feature gathering and multilayer perceptron

**DOI:** 10.3389/fpls.2024.1344026

**Published:** 2024-01-12

**Authors:** Fan Wang, Linghan Song, Xiaojie Liu, Shuangwen Zhong, Jiawei Wang, Yao Zhang, Yun Wu

**Affiliations:** ^1^ College of Forestry, Fujian Agriculture and Forestry University, Fuzhou, Fujian, China; ^2^ University Key Lab for Geomatics Technology and Optimize Resource Utilization in Fujian Province, Fujian Agriculture and Forestry University, Fuzhou, Fujian, China

**Keywords:** UAV-based remote sensing, spectrum-spatial fusion, three-dimensional spectral, deep learning, masson pine forest

In the published article, there was an error in the author list; author Fan Wang has been corrected from last author to first author. The corrected author list appears below.

Fan Wang^1,2 * †^, Linghan Song ^1,2 †^, Xiaojie Liu^1,2^, Shuangwen Zhong^1,2^, Jiawei Wang^1,2^, Yao Zhang^1,2^ and Yun Wu^1,2^

The authors apologize for this error and state that this does not change the scientific conclusions of the article in any way. The original article has been updated.

